# Hyperspectral Reflectance of Light-Adapted Leaves Can Predict Both Dark- and Light-Adapted Chl Fluorescence Parameters, and the Effects of Chronic Ozone Exposure on Date Palm (*Phoenix dactylifera*)

**DOI:** 10.3390/ijms21176441

**Published:** 2020-09-03

**Authors:** Lorenzo Cotrozzi, Giacomo Lorenzini, Cristina Nali, Elisa Pellegrini, Vincenzo Saponaro, Yasutomo Hoshika, Leila Arab, Heinz Rennenberg, Elena Paoletti

**Affiliations:** 1 Department of Agriculture, Food and Environment, University of Pisa, Via del Borghetto 80, 56124 Pisa, Italy; lorenzo.cotrozzi@agr.unipi.it (L.C.); giacomo.lorenzini@unipi.it (G.L.); cristina.nali@unipi.it (C.N.); vincenzo.saponaroo@gmail.com (V.S.); 2 Institute of Research on Terrestrial Ecosystems, National Research Council of Italy, Via Madonna del Piano 10, 50019 Sesto Fiorentino, Florence, Italy; yasutomo.hoshika@cnr.it (Y.H.); elena.paoletti@cnr.it (E.P.); 3 Chair of Tree Physiology, Institute of Forest Sciences, Albert-Ludwigs-University Freiburg, Georges-Köhler-Allee 53/54, 79110 Freiburg, Germany; leila.arab@ctp.uni-freiburg.de (L.A.); heinz.rennenberg@ctp.uni-freiburg.de (H.R.)

**Keywords:** Fluorescence quenching, hyperspectral phenotyping, partial least squares regression, photosystem II activity, pulse-amplitude-modulation fluorometry, saturation pulse method, spectral signature, vegetation index, vegetation spectroscopy

## Abstract

High-throughput and large-scale measurements of chlorophyll *a* fluorescence (ChlF) are of great interest to investigate the photosynthetic performance of plants in the field. Here, we tested the capability to rapidly, precisely, and simultaneously estimate the number of pulse-amplitude-modulation ChlF parameters commonly calculated from both dark- and light-adapted leaves (an operation which usually takes tens of minutes) from the reflectance of hyperspectral data collected on light-adapted leaves of date palm seedlings chronically exposed in a FACE facility to three ozone (O_3_) concentrations (ambient air, AA; target 1.5 × AA O_3_, named as moderate O_3_, MO; target 2 × AA O_3_, named as elevated O_3_, EO) for 75 consecutive days. Leaf spectral measurements were paired with reference measurements of ChlF, and predictive spectral models were constructed using partial least squares regression. Most of the ChlF parameters were well predicted by spectroscopic models (average model goodness-of-fit for validation, *R^2^*: 0.53–0.82). Furthermore, comparing the full-range spectral profiles (i.e., 400–2400 nm), it was possible to distinguish with high accuracy (81% of success) plants exposed to the different O_3_ concentrations, especially those exposed to EO from those exposed to MO and AA. This was possible even in the absence of visible foliar injury and using a moderately O_3_-susceptible species like the date palm. The latter view is confirmed by the few variations of the ChlF parameters, that occurred only under EO. The results of the current study could be applied in several scientific fields, such as precision agriculture and plant phenotyping.

## 1. Introduction

No investigation into the photosynthetic performance of plants seems complete without chlorophyll *a* fluorescence (ChlF) data [1]. Thanks to the introduction of a number of highly user-friendly and portable chlorophyll fluorometers, ChlF—a non-invasive and relatively low-cost measurement of photosystem II (PSII) activity—has become one of the most powerful and used techniques available to plant physiologists and ecophysiologists in the last 30 years [1]. The sensitivity of PSII activity to abiotic and biotic stressors has made this approach a key technique not only for understanding the photosynthetic mechanisms but also as a broader indicator of how plants respond to environmental change, although its ineffectiveness has also been reported sometimes [2]. Among the available types of chlorophyll fluorometers, the pulse-amplitude-modulation (PAM) fluorometry, in conjunction with the saturation pulse method, remains the most utilized approach [2,3]. Briefly, this method consists of adapting a leaf to the dark until all the reaction centers are open, and then expose the leaf to light. This gives rise (usually for a few seconds) to a progressive closure of PSII reaction centers, resulting in an increase in the yield of ChlF. Thereafter, the fluorescence level typically starts to fall again through a phenomenon termed “fluorescence quenching”, and explained in two ways: (i) an increase in the rate by which electrons are transported away from PSII due to the light-induced activation of enzymes involved in carbon metabolism and the opening of stomata (i.e., ‘photochemical quenching’), and (ii) an increase in the efficiency by which energy is converted to heat (i.e., ‘non-photochemical quenching’) [1]. A large number of coefficients have been calculated to quantify photochemical and non-photochemical quenching, as reported in excellent reviews (e.g., [1,2,4]).

Collection of both dark- and light-adapted measurements to calculate all ChlF parameters may take a minimum of 20 min for each dark-adapted leaf, and other several minutes or much longer (e.g., >15 min) to achieve a light-adapted steady-state (starting from a dark-adapted state). As a consequence, it is rarely possible to go through an entire quenching protocol, especially in the field due to time and weather constraints [2]. For this reason, together with the great interest of field physiologists in high-throughput and large-scale measurements of ChlF [2], new less time-consuming techniques (e.g., imaging fluorescence, long-term monitoring fluorescence, remote sensing of ‘passive’ fluorescence) are under continual development, although substantial limitations remain in their use (e.g., instrumentation reliability and data interpretation [5,6,7]).

Vegetation spectroscopy is a high-throughput sensor technology based on the optical properties of living vegetation (e.g., leaf and canopy reflectance) that enables the rapid and non-destructive assessment of plant status, along with a simultaneous estimate of several plant traits in the field on a large number of plants over multiple time periods [8]. The prediction of these traits from leaf spectra is based on the exploitation of the relationships of light with molecular organic bonds, mainly C-H, N-H, and O-H, resulting in vibrational excitation at specific wavelengths through the visible (VIS: 400–700 nm), the near-infrared (NIR: 700–1100 nm) and the short-wave infrared (SWIR: 1100–2400 nm) spectral regions [9]. Several simple vegetation indices (VIs) based on the ratio of reflected light at different wavelengths have been developed because related to various plant traits (e.g., normalized difference vegetation index, NDVI [10], or chlorophyll index, CI [11]). Another ever-expanding approach regards the use of multivariate methods to directly model commonly used plant traits as a function of the spectral profiles. Advances in the sensitivity and portability of hyperspectral spectrometers, as well as in computational capacity and chemometric modeling (e.g., using partial least squares regression, PLSR [12]), have enabled the use of this approach to estimate a variety of commonly investigated plant traits and physiological processes based on foliar optical properties, including morphological, physiological, and biochemical parameters (e.g., [13,14,15,16,17]). The model calibration is accomplished by pairing leaf spectra, collected using a uniform and stable light source in a consistent manner, with independent and reliable reference measurements. Subsequently, the model is validated by comparing relationships between observed and predicted values collected on other independent samples. This calibration model can then be used to predict the variable of interest in unknown samples on the basis of their spectral signature alone [18]. Despite the similarities of vegetation spectroscopy and ChlF measurements, and some attempts to evaluate relations between some VIs (i.e., using a few wavelengths) and ChlF parameters (e.g., [19,20]), to the best of our knowledge, no study has examined the ability of reflectance profiles to directly estimate widely-used PAM ChlF parameters by using a multivariate modeling approach on hyperspectral data (i.e., using many wavelengths). 

Date palm (*Phoenix dactilyfera* L.) is among the first crops domesticated by early human civilization, and over 100 million date palms live in the world. This perennial, dioecious plant of the Arecaceae family, plays an important nutritional, social, environmental, cultural, and economic role for many people living in arid and semiarid regions of North Africa and the middle east. Within the last centuries, it has been introduced to Southeastern Asia, Southern Africa, Australia, South America, Mexico, and the United States [21,22]. Date palm is exceptional in the sense that it can withstand extreme temperatures and drought [23,24,25,26], as well as other harsh climatic conditions, such as episodes of high tropospheric O_3_ concentration [27]. Actually, although there is a high interest in exploring the responses of date palms to O_3_ exposure, this experiment and related papers on growth, physiological and biochemical responses (e.g., [28]) is the first one to investigate the effects of chronic O_3_ exposure on this species.

Tropospheric O_3_ is a major phytotoxic air pollutant produced photochemically by a variety of precursors, such as nitrogen oxides and volatile organic compounds, with harmful effects also on biota, including plant and animal health [29,30,31]. Levels of O_3_ are still elevated in many areas of the world such as Europe, North America, and Southeastern Asia, and are predicted to raise further due to the occurrence of climatic changes and to anthropogenic pressures [32], especially in hot-spot regions such as the Arabian Peninsula and the Mediterranean area [27,33]. However, reductions in tropospheric O_3_ concentrations have also been reported in some Mediterranean sites [34]. Excessive O_3_ uptake by plants induces detrimental effects such as accelerated leaf senescence, reduction of photosynthesis and growth, partial stomatal closure, cell dehydration, cell destruction by excessive excitation energy, and appearance of chlorotic/necrotic leaf injuries, that overall result in the reduction of plant yield both in terms of quantity and quality and huge economic losses [29,35].

The use of ChlF as a suitable method for studying the responses of plants to O_3_ (as well as to other abiotic and biotic stress factors) has been reported for long (e.g., [36,37,38]). In this study, we tested the capability to rapidly, precisely, and simultaneously estimate the number of PAM ChlF parameters commonly calculated from both dark- and light-adapted leaves from reflectance hyperspectral data collected on light-adapted leaves of date palm seedlings chronically exposed to three levels of O_3_ in an O_3_-Free Air Controlled Exposure (FACE) facility located in the Mediterranean environment (i.e., Italy). Furthermore, since spectra themselves are a phenotypic expression of the aggregate signals of chemical, morphological and physiological properties of leaves under specific environmental conditions [8], we evaluated the potential of full-range (400–2400 nm) hyperspectral phenotyping to pre-visually, and accurately detect and classify the environmental pressures induced by the gradient of O_3_ concentrations on date palm, and whether these effects are in accordance with O_3_-induced variations of ChlF parameters, and other VIs estimated from spectra, thus also investigating the response of this species to chronic O_3_ pollution.

## 2. Results

### 2.1. Predictions of ChlF Parameters

Various spectral ranges (characterized by specific absorption features known in the literature that we thought would directly or indirectly relate to specific traits), and number of components (Table S1) were firstly investigated to get best prediction accuracy [i.e., highest model goodness-of-fit (*R^2^*) and lowest bias, root mean square error (RMSE), and percent RMSE of the data range (%_RMSE_)] of the PLSR models developed for the estimation of the ChlF parameters (i.e., F_0_, F_m_, F_v_/F_m_, F_s_, F_m_’, F_0_′, Φ_PSII_, F_v_’/F_m_’, ETR, qP, qN, NPQ, qL, P, D; see Section 4.2 for parameter descriptions). Most of the final models utilized the wavelength range 600–900 nm and included 6–8 components (i.e., F_0_, F_m_, F_v_/F_m_, F_0_^′^, Φ_PSII_, F_v_’/F_m_’, qP, qN, NPQ, qL, D). The final F_s_ and F_m_’ PLSR models utilized the 400–1200 nm spectral region, including four and eight components, respectively. The full range 400–2400 nm was utilized in the final PLSR models for predictions of ETR and P, including 12 components for both of them (Table 1). 

**Figure 1:**
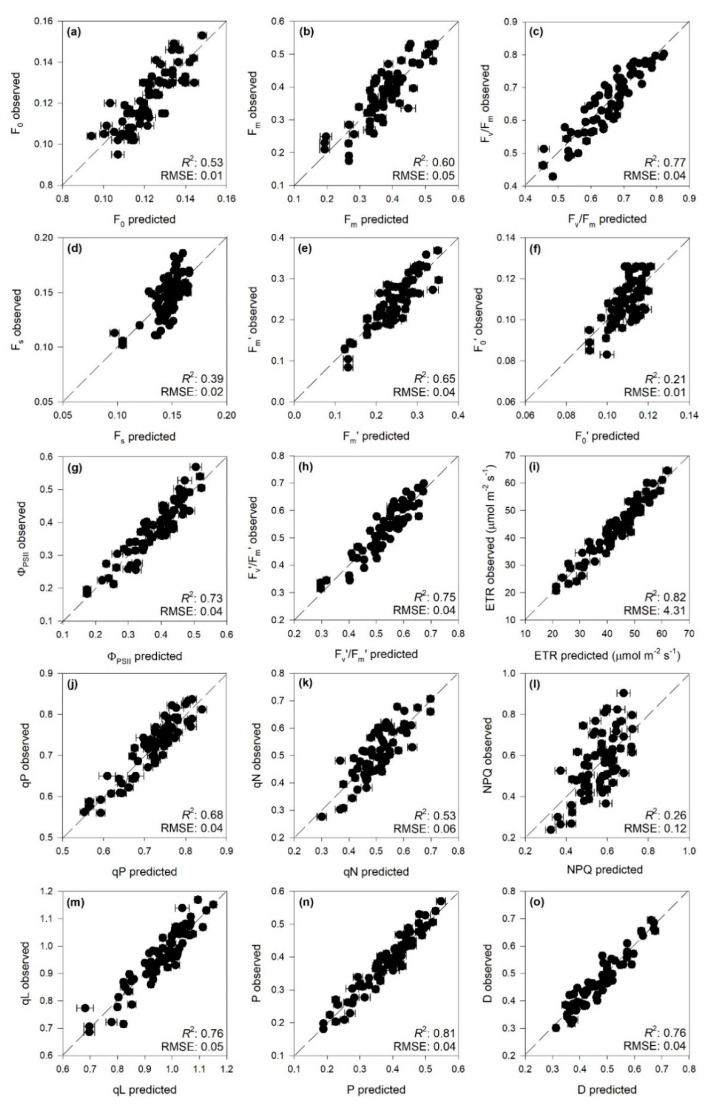
Observed vs. partial least squares regression (PLSR)-predicted values of chlorophyll *a* fluorescence parameters in date palm leaves; error bars for predicted values represent the standard deviation generated from 500 simulated models; dashed line is 1:1 relationship; model goodness-fit (*R^2^*), and root mean square error (RMSE) for validation data generated using 80% of the data for calibration and 20% for validation are reported. Bias outputs are not shown as they were always lower than 0.01. (**a**) F_0_; (**b**) F_m_; (**c**) F_v_/F_m_; (**d**) F_s_; (**e**) F_m_’; (**f**) F_0_′; (**g**) Φ_PSII_; (**h**) F_v_’/F_m_’; (**i**) ETR; (**j**) qP; (**k**) qN; (**l**) NPQ; (**m**) qL; (**n**) P; (**o**) D. See Table 1 caption for the parameter abbreviations.

PLSR models very accurately characterized F_v_/F_m_, Φ_PSII_, F_v_’/F_m_’, ETR, qP, qL, P, and D (*R^2^* and %_RMSE_ for validation: 0.77 and 11%, 0.73 and 11%, 0.75 and 11%, 0.82 and 10%, 0.68 and 14%, 0.76 and 11%, 0.81 and 10%, 0.76 and 10%, respectively). High prediction performance was also found for F_0_, F_m_, F_m_’, and qN (*R^2^* and %_RMSE_ for validation: 0.53 and 17%, 0.60 and 12%, 0.65 and 12%, 0.53 and 15%), whereas low prediction accuracy was found for F_s_, F_0_^′^, and NPQ (Table 1 and Figure 1). For both calibration and validation, bias was always lower than 0.01. Among the PLSR models with good prediction accuracy, fit statistics for external validations were consistent with those registered for the validations, except for F_m_, F_m_’, F_v_’/F_m_’, and qL that were lower (Table S2).

**Figure 2:**
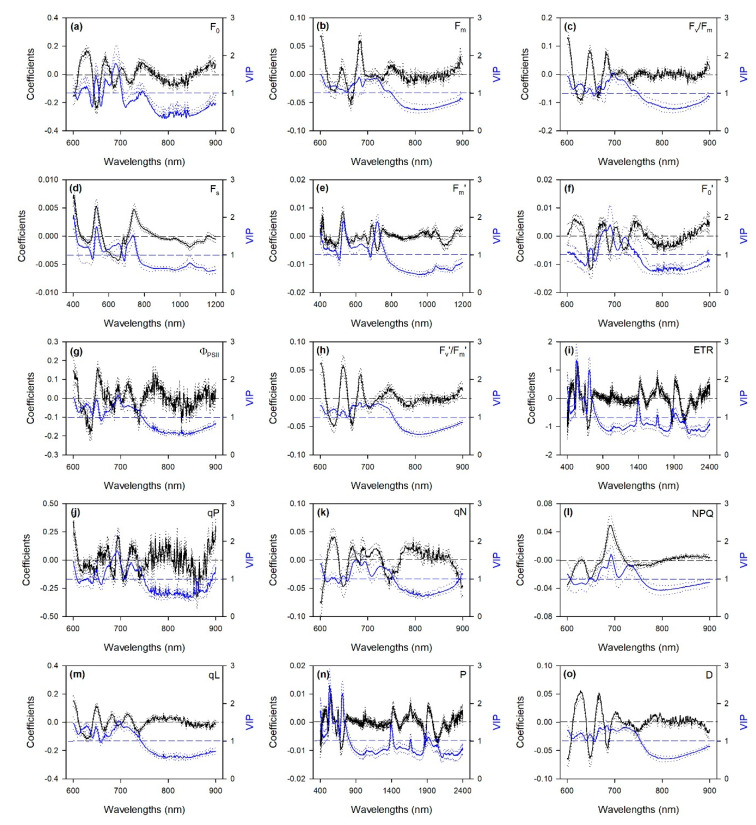
Mean (solid), 5^th^ and 95^th^ percentile (dotted) of standardized coefficients (black) and variable importance for projection (VIP, blue) by wavelengths for the PLSR models predicting chlorophyll *a* fluorescence parameters in date palm leaves. (**a**) F_0_; (**b**) F_m_; (**c**) F_v_/F_m_; (**d**) F_s_; (**e**) F_m_’; (**f**) F_0_′; (**g**) Φ_PSII_; (**h**) F_v_’/F_m_’; (**i**) ETR; (**j**) qP; (**k**) qN; (**l**) NPQ; (**m**) qL; (**n**) P; (**o**) D. See Table 1 caption for parameter abbreviations.

Profiles of standardized coefficients (i.e., centered and scaled) and variable importance to the projection (VIP) metrics from PLSR models using the 600–900 nm spectral range (i.e., those for the predictions of F_0_, F_m_, F_v_/F_m_, F_0_^′^, Φ_PSII_, F_v_’/F_m_’, qP, qN, NPQ, qL, and D) highlighted the 600–750 nm wavelengths as particularly important for estimations, especially peaking around 650, 680–690 and 700–750 nm (Figure 2). Standardized coefficients and VIP values of PLSR models, including the 400–1200 nm wavelength range (i.e., those for the estimations of F_s_ and F_m_’) highlighted not only important spectral wavelengths from 650 to 750 nm, but also those that peaked around 420 and 500–550 nm. Peaks from 450 to 750 nm were also observed in profiles of standardized coefficients and VIP of PLSR models using the full range (i.e., those for the predictions of ETR and P), but here other peaks were also observed around 1400, 1700, and 1900–1950 nm.

### 2.2. Hyperspectral Phenotyping

**Figure 3:**
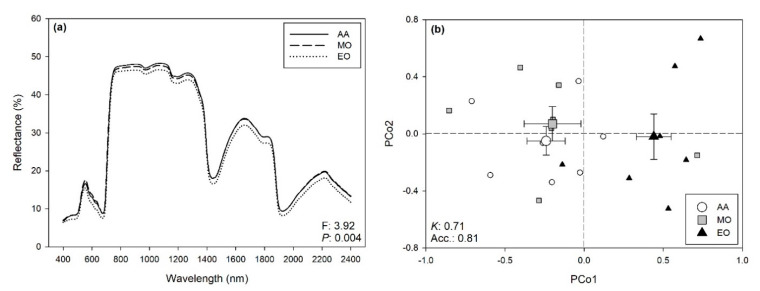
(**a**) Average foliar reflectance profiles of date palms exposed to ambient air (AA), moderate ozone (MO), and elevated ozone (EO); F- and *P*-values from permutational analysis of variance (PERMANOVA) for the effects of ozone on the full range (400–2400 nm) reflectance profiles of date palm leaves are reported in the bottom-right corner of the panel. (**b**) Scores (mean ± standard error) for the first and second principal components from principal coordinates analysis (PCoA) of the reflectance data (400–2400 nm) collected from date palm leaves, highlighting the capability of spectroscopy to discriminate plants exposed to AA (white circle) vs. plants exposed to MO (gray square) and EO (black triangle); average accuracy (Acc.) and *Kappa* (*K*) values from partial least squares discriminant analysis (PLS-DA) are reported on the bottom-left corner of the panel.

Multiple different spectral ranges were initially investigated to optimize the statistical outputs of the permutational analysis of variance (PERMANOVA; Table S1), and the best outputs were finally recorded using the full range (i.e., 400–2400 nm). Although no visible symptoms were observed on the leaves, PERMANOVA revealed that O_3_ exposure affected the reflectance profile of the date palm (F: 3.92, *P*: 0.004; Figure 3a), as well shown by Figure 3b, which summarizes the outputs of the principal coordinates analysis (PCoA). Best classifications of O_3_ treatments from the spectra (i.e., higher mean *Kappa*) by partial least squares discriminant analysis (PLS-DA) were found with an 80:20 ratio for calibration:validation data, using 13 components: *Kappa*, and the Accuracy for the validation were 0.71 ± 0.20 and 0.81 ± 0.13 (mean ± standard deviation), respectively. Specifically, elevated O_3_ (EO) was accurately discriminated from the other O_3_ treatments, while misclassifications occurred between the ambient air (AA) and moderate O_3_ (MO) treatments (Figure 3b, Table S3).

### 2.3. Variations of Spectra-Estimated ChlF Parameters and VIs

**Figure 4:**
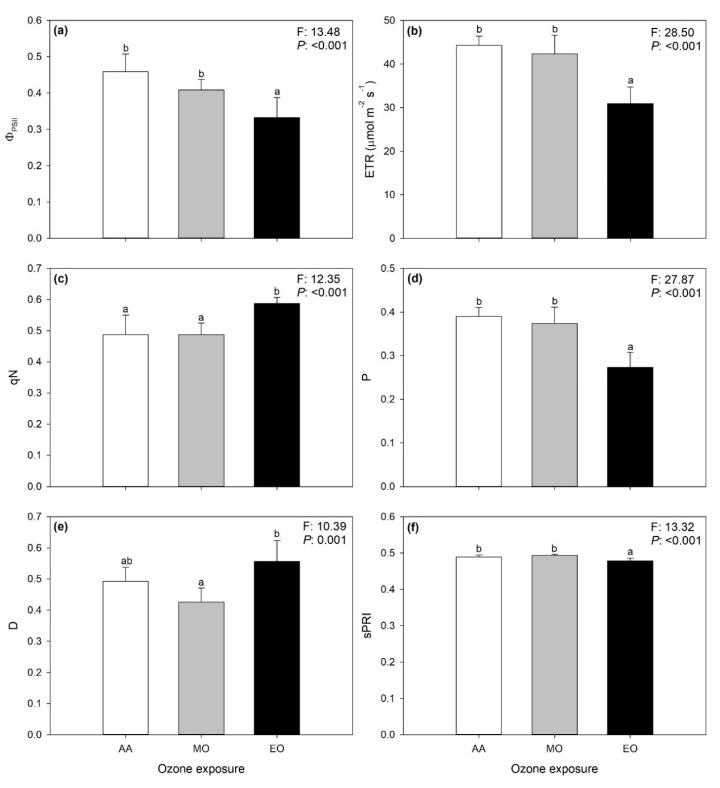
Variations of PSII operating efficiency in light conditions, Φ_PSII_ (**a**); ETR, electron transport rate, ETR (**b**); non-photochemical quenching calculated as (F_m_ − F_m_’)/(F_m_ − F_0_′), qN (**c**); fraction of light absorbed in PSII antennae that is utilized in PSII photochemistry, P (**d**); fraction of light absorbed in PSII antennae that is dissipated thermally, D (**e**); and (scaled) photosynthetic reflectance index, sPRI (**f**) estimated from spectra collected on leaves of date palm under ambient (AA, white), moderate (MO, gray) and elevate (EO, black) ozone concentrations. Data are shown as mean ± standard deviation. F- and *p*-values for the effects of ozone from a one-way ANOVA are shown in the top-right corner of panels. According to Tukey’s post-hoc test, different letters indicate significant differences among means (*p*: ≤0.05).

The significant effects of O_3_ on VIs and selected ChlF parameters (i.e., those for whom the PLSR models showed *R^2^* ≥ 0.55 for external validation) estimated from the spectra are shown in Figure 4. Φ_PSII_, ETR, P, and photochemical reflectance index (scaled; sPRI) decreased only under elevated ozone (EO; −27, −30, −30, and −2% compared with ambient air, AA, respectively), whereas qN increased only under EO (+21%). Although D values were higher under EO than under moderate ozone (MO), no significant differences were observed among plants exposed to AA and those exposed to higher O_3_ concentrations. No significant O_3_ effects were observed on F_v_/F_m_, qP, plant senescence reflectance index (scaled; sPSRI), and CI (*p* > 0.05).

## 3. Discussion

This study shows the ability of reflectance spectroscopy to rapidly, accurately, and non-destructively monitor the sensitivity of PSII efficiency to environmental constraints, specifically to O_3_ pollution. By combining hyperspectral reflectance collections, standard PAM ChlF measurements, and robust statistical modeling, this study demonstrated the potential to concomitantly predict from spectra of light-adapted leaves of date palm a number of ChlF parameters that are commonly collected from measurements of both dark- and light-adapted leaves to investigate the photosynthetic performance of plants under abiotic and biotic stressors.

Standard collections of the whole set of PAM ChlF parameters are often logistically challenging, usually requiring several minutes per leaf as the leaf has first to reach the dark-adapted state and then come back to the light-adapted state [2]. Spectral approaches have been shown as a valid alternative to standard measurements of photosynthetic activity in plants since they are able to estimate photosynthetic processes through VIs correlated with photosynthetic processes (e.g., the xanthophyll cycle by PRI [39,40]). In addition, specific and commonly used photosynthetic traits such as net photosynthesis, stomatal conductance, the maximum rate of carboxylation, and the maximum rate of electron transport [17,41,42,43,44] could directly be predicted from spectral data. Some attempts to evaluate the relations between VIs and a few ChlF parameters have also been reported (e.g., [19,20]). However, to our knowledge, this is the first study to document the potential of vegetation spectroscopy to directly estimate widely used ChlF parameters.

We found an excellent prediction performance for most of the PLSR modeled ChlF parameters. F_v_/F_m_, Φ_PSII_, F_v_’/F_m_’, ETR, qP, qL, P and D were estimated with very high accuracy (validation *R^2^* 0.68–0.82), and the PLSR prediction approach resulted in more precise data than previous efforts performed through relations between VIs and some ChlF parameters (i.e., F_v_/F_m_ and F_v_’/F_m_’; e.g., [20]). Good prediction performance was also found for F_0_, F_m_, F_m_’, and qN (validation *R^2^*: 0.53–0.65). However, we encourage more caution when interpreting results from a narrow range of values of these parameters, since the PLSR models we developed may have limitations by discriminating fine scale differences. Low accuracies were instead reported only for F_s_, F_0_′, and NPQ. Undoubtedly, a novel outcome of these results is the ability to rapidly, precisely, and simultaneously estimate a number of ChlF parameters commonly calculated from both dark- and light-adapted leaves from reflectance hyperspectral data collected on light-adapted leaves. This was likely possible because ChlF parameters that also require dark-adapted leaves, describe PSII mechanisms that are related to the foliar properties expressed in the leaf spectrum collected under the light-adapted state. It is also interesting to note that none of the ChlF parameters representing punctual fluorescence intensity at a specific time and light condition (i.e., F_0_, F_m_, F_m_’, F_s_, and F_0_′) was among the parameters estimated with high accuracy. This might be due to an inability of leaf spectra to describe a specific fluorescence emission, as opposed to their ability to summarize plant features related to the PSII photochemistry described by F_v_/F_m_, Φ_PSII_, F_v_’/F_m_’, ETR, qP, qL, P, and D. Another explanation might be found in the smaller ranges observed for F_0_, F_m_, F_m_’, F_s_, and F_0_′ values compared with other parameters, since PLSR models would work better when the calibration is accomplished by pairing spectral data with the observed traits having a greater proportion of variation. Furthermore, even if we might have expected complexity in the prediction from leaf spectra of a phenomenon like the photoprotective thermal dissipation of excess excitation energy (i.e., non-photochemical quenching), the reason for differences in the prediction accuracy between qN and NPQ remains an open question. The drop-in prediction accuracy observed for the external validation of a few PLSR modeled parameters (compared with PLSR validation) was instead likely due to the scarcity of available samples usable for external validation. However, this is just one of many points that require further research on the topic to be addressed.

Nevertheless, it is not surprising that best predictions of most of the modeled ChlF parameters were obtained using only the wavelengths from 600 to 900 nm (this also means that these parameters could be estimable by inexpensive optical instrumentation [9]). The use of narrower ranges, including only specific absorption wavelengths for the trait to be estimated, sometimes leads to better predictions than using wider ranges, since the incorporation of other spectral regions may reduce the prediction ability of trait-specific wavelengths [15,17]. Indeed, the 600–900 nm spectral range finally used for estimations of F_0_, F_m_, F_v_/F_m_, F_0_′, Φ_PSII_, F_v_’/F_m_’, qP, qN, NPQ, qL, and D includes the red chlorophyll absorption peaks around 700 nm [45], the whole re-emission ChlF spectrum of a leaf ranging from 650 to 800 nm [46], as well as the red-edge at 700–750 nm [47]. The importance of this pigment-related spectral region in the assessment of photosynthetic processes has been largely shown for several plant species (e.g., [39,41,43,45]). A number of studies have also reported that the shape of the red-edge is dependent on chlorophyll content (e.g., [48,49]) and stress conditions (e.g., [9,47]). This supports the importance of wavelengths around 650, 680–690, and 700–750 nm in predicting F_0_, F_m_, F_v_/F_m_, F_0_′, Φ_PSII_, F_v_’/F_m_’, qP, qN, NPQ, qL, and D highlighted by their standardized coefficient and VIP profiles. Standardized coefficients and VIP values of F_s_ and F_m_’ PLSR models, using the VIS-NIR (i.e., 400–1200 nm) spectral range, also highlighted the importance of including wavelengths around 420 and 500–550 nm in the prediction of these parameters, thus also containing the blue peaks of chlorophylls as well as of carotenoids [45]. Conversely, since using wider ranges means the incorporation of more signals of chemical, morphological, and physiological properties of leaves included in the spectra [8], ETR and P values were better predicted using the full range (i.e., 400–2400 nm), thus including other absorbance features contained outside the pigment-related spectral range. Effectively, profiles of standardized coefficients and the VIP of PLSR models of these traits also peaked around 1400, 1700, and 1900–1950 nm, which are well-known water and protein absorption features [50], suggesting that the variations of these compounds somehow helped the estimation of ETR and P from spectra.

The present study also evidenced that we were able to discriminate with high accuracy (81% of success) the effects of O_3_ exposure on reflectance profiles of the date palm leaves. Specifically, even in the absence of visible foliar symptoms, we were able to discriminate plants exposed to the higher O_3_ concentration (i.e., EO) from those exposed to the lower O_3_ levels, while a certain misclassification occurred between samples exposed to AA and MO. This might be due to a moderate O_3_ susceptibility of date palm, but better outputs might be reached by raising the experimental/plant replications adopted for the hyperspectral phenotyping (this is especially true under field conditions, usually characterized by highly variable growth environments). On the one hand, these results confirm the capability of this approach (i.e., hyperspectral phenotyping through the analyses of spectral signatures) to detect O_3_ effects on plants, as previously reported for various other abiotic and for biotic stressors (e.g., [8,17,51,52]). On the other hand, the present results confirm that the efficiency of this spectral approach is dependent on the sensitivity of the plants/cultivars to O_3_, as well as to the magnitude at which this environmental pressure is imposed on vegetation.

The phytotoxicological outcomes of the analyses of spectral signatures were confirmed by variations of the investigated ChlF parameters derived from spectra (again, only parameters from best performing PLSR models were used) and VIs, further confirming the potential of vegetation spectroscopy in monitoring the responses of plants to O_3_. Although increased O_3_ concentrations did not induce photoinhibitory damage and early senescence or altered chlorophyll levels (i.e., unchanged F_v_/F_m_, qP, sPSRI and CI), EO caused a reduction of PSII performance (i.e., reduced ΦPSII, ETR, and P), together with an activation of the dissipation of the excess excitation energy as heat (i.e., increased qN and sPRI; and higher D values under EO than under MO, although no significant differences with AA). Although further molecular, biochemical, physiological, and morphological investigations are needed to properly investigate the interaction of date palm with O_3_, the results reported in the present study suggest an ability of the date palm to tolerate moderate O_3_ pollution levels, in accordance with previous reports highlighting the high capability of this ancient plant species to withstand extreme and harsh climatic conditions (e.g., [23,24,25,26]).

In conclusion, the present study firstly shows that vegetation spectroscopy can be a rapid, non-destructive, and relatively inexpensive tool to concomitantly and accurately estimate an array of widely-used ChlF parameters, using a single spectral measurement. This result suggests that the prediction from spectral data can be used in standard reference measurements, thus allowing a dramatic increase of ChlF data collection from a larger number of individual genotypes and plants, than reference measurements alone. Second, this study confirms that hyperspectral information can successfully detect stress conditions in plants exposed to O_3_, even in the absence of visible foliar symptoms and using a moderately O_3_-susceptible species like the date palm, as suggested by the few variations of the investigated ChlF parameters and VIs. It would be interesting to perform further investigations and validations of the proposed approach on other species under heterogeneous environments.

The results presented in the current study could be applied in a number of scientific fields such as precision agriculture and high-throughput plant phenotyping and could provide significant benefits to increase our knowledge on the intimate mechanisms of oxidative stress in plant cells, so to achieve better plant yield and quality. Furthermore, while logistical challenges (e.g., solar angle or atmospheric interference) exist, leaf-level spectroscopy, as adopted in the present study, can be effectively used as a ground reference or training input for airborne-based platforms, and scaled to field and landscape levels [13,53].

## 4. Materials and Methods 

### 4.1. Plant Material and Experimental Design

On December 2018, micro-propagated one-year-old plants of *P. dactylifera* cv Nabut Saif were purchased from a commercial nursery (Date Palm Developments Ltd., Sommerset, U.K.) and directly planted in 4.5 L pots containing gravel (∅ 3–6 mm) and commercial planting peat-rich soil (70:30 in volume). Pots were kept to overwinter in a phytotron on plastic tablets continuously filled with tap water at 1–2 cm height and watered daily with 50 mL tap water per plot. Controlled conditions included 25 °C temperature and artificial illumination at around 200 µmol m^−2^ s^−1^ with a 16 h of photoperiod. On the 1st of May 2019, plants were transferred into 20-L pots containing the same substrate and maintained well-irrigated under shaded tunnels under field conditions, and moved to full light after one week into the O_3_-FACE facility of Sesto Fiorentino, Florence, Italy (43°48′59′’ N, 11°12′01′’ E, 55 m a.s.l.). Here, uniform-sized plants (around 1 m tall) were selected and exposed to three levels of O_3_ (target concentrations: 1.0, 1.5 and 2.0 times the ambient air level) named as ambient air (AA), moderate O_3_ (MO) and elevated O_3_ (EO); treatments lasted from the 20th of May to the 2nd of August 2019, for 75 consecutive days (i.e., around 2.5 months). Three replicated 5 × 5 × 2 m (L × W × H) plots were assigned to each O_3_ treatment, with five plants per each plot. Each pot was fertilized once a month with NPK 20:10:20 with micronutrients (Soluplant 20.10.20, Haifa, Bologna, Italy). Each pot was watered daily by a drip system with 200 mL of tap water (i.e., 90% of field capacity). A detailed description of the facility and of O_3_ exposure methodology is reported in Paoletti et al. [54]. The maximum hourly O_3_ concentrations throughout the whole experimental period were 110.2 ppb in AA, 169.4 ppb in MO and 183.2 ppb in EO, respectively; while the Accumulated exposure Over Threshold of 40 ppb (AOT40) values resulted of 16.1 ppm h, 36.9 ppm h and 50.1 ppm h, respectively.

The collection of ChlF measurements and leaf spectra was performed from the 30^th^ of July to the 2nd of August 2019 (i.e., four consecutive days). The youngest mature leaves (the 4^th^ or the 5^th^ counting from the top) of each plant were selected, and measurements were performed on the 4^th^ leaflets (counting from the apex of the leaf) located on the right side of the rachis, looking at the adaxial leaf surface. Every day, around 25 plants, randomly distributed across O_3_ treatments, and the experimetal plots were measured from 9.00 to 16.00, with a total of 102 samples collected. These combined spectral and standard measurements were used to build multivariate methods to predict ChlF parameters from spectral data (see below). Furthermore, on the last day of measurements, eight plants per O_3_ treatment were randomly selected, and consecutively measured only for leaf reflectance within the shortest possible time (i.e., around 20 min). These reflectance measurements were used for the analyses of spectral signatures (i.e., hyperspectral phenotyping), and for the final estimations of vegetation indexes and other leaf traits by the PLSR models developed in this study (see below).

### 4.2. Chlorophyll a Fluorescence

Chlorophyll a fluorescence measurements were collected using a PAM-2000 fluorometer (Walz, Effeltrich, Germany). The minimum and maximum fluorescence yield in the dark-adapted state (F_0_ and F_m_, respectively) were determined immediately before and after the application of a 0.8 s saturating light pulse at 8000 µmol m^−2^ s^−1^ in 40 min dark-adapted leaves. An actinic light at around 270 μmol m^−2^ s^−1^ was then turned on, and maximum fluorescence intensity in the light-adapted state (F_m_’) was determined applying saturating light pulses every 20 s, whereas minimum fluorescence intensity in the light-adapted state (F_0_′) was determined immediately after turning off the actinic source in the presence of a far-red (>710 nm) background for 3 s to ensure maximal oxidation of PSII electron acceptors. Both F_m_’ and F_0_′ were recorded 10 min after the turning on of the actinic light, when fluorescence reached a steady-state intensity in a light-adapted state (F_s_).

The maximum quantum efficiency of PSII photochemistry was calculated as F_v_/F_m_ = (F_m_ − F_0_)/F_m_, whereas the PSII operating efficiency in light conditions was calculated as Φ_PSII_ = (F_m_’ − F_s_)/F_m_’ [55]. The maximum efficiency of PSII in light conditions was calculated as F_v_’/F_m_’ = (F_m_’ − F_0′_)/F_m_’ [56]. The electron transport rate was calculated as ETR = Φ_PSII_ × photosynthetic photon flux density (PPFD) × 0.5 × 0.84, where 0.5 is the partitioning of absorbed quanta between PSII and photosystem I (PSI) and 0.84 is the absorption coefficient of an average leaf [57]. The photochemical quenching was calculated as qP = (F_m_’ − F_s_)/(F_m_’ − F_0_′), whereas the non-photochemical quenching was calculated as qN = (F_m_ − F_m_’)/(F_m_ − F_0_′) and as NPQ = (F_m_ − F_m_’)/F_m_’ [58]. The fraction of open PSII centers was estimated as qL = qP × (F_0_′/F_s_) [59]. The fraction of light absorbed in the PSII antennae that is utilized in the PSII photochemistry was estimated as P = (F_v_’/F_m_’) × qP, whereas the fraction of light absorbed in the PSII antennae that is dissipated thermally was estimated as D = 1 − F_v_’/F_m_’ [56]. Further details about the ChlF parameters are reported in several reviews (e.g., [2,3,4]).

### 4.3. Hyperspectral Reflectance

The full range (350–2500 nm) leaf reflectance profiles were collected by an ASD FieldSpec 4 spectroradiometer (Analytical Spectral Devices, Boulder, CO, USA), using a leaf clip with an internal halogen light source attached to a plant probe. The measurements were acquired on four areas (randomly selected, ∅ 1 cm) of the adaxial surface of each leaf, with one measurement per area, and the collections were combined to produce an average leaf spectrum. The relative reflectance of each leaf was determined from the measurement of the leaf radiance divided by the radiance of a white reference panel included in the leaf clip, measured before each plant (i.e., four spectral collections) when collecting spectra for modeling, or every four plants (i.e., 16 spectral collections) when collecting spectra for hyperspectral phenotyping.

### 4.4. PLSR-model Calibration and Validations

The above-mentioned ChlF parameters (i.e., F_0_, F_m_, F_v_/F_m_, F_s_, F_m_’, F_0_′, Φ_PSII_, F_v_’/F_m_’, ETR, qP, qN, NPQ, qL, P, D) were predicted from spectra using a PLSR [12] modeling approach, using untransformed reflectance profiles (only spectral jump correction and data interpolation were performed). This “spectra-trait” modeling was performed using ca. 80% of the whole dataset, in order to also allow for an external validation of the developed PLSR models (see below). PLSR modeling has become the favorite chemometric approach (e.g., [14,15,16,60]) because, conversely to classical regression techniques, it reduces a large number of collinear predictor variables (as in the case of hyperspectral data) to relatively few, uncorrelated latent variables, avoiding the risk of producing unreliable coefficients and error estimates [61]. To avoid potential model overfitting, the numbers of the latent variables used were selected based on the reduction of the predicted residual sum of squares (PRESS) statistics [62] by leave-one-out cross-validation. Finally, the selected sets of extracted components were combined with linear models predicting ChlF parameters from leaf reflectance profiles.

The model performance was assessed by running 500 randomized permutations of the datasets using 80% of the data for calibration and the remaining 20% for validation (i.e., internal validation). The *R^2^*, RMSE, bias, and %RMSE of both the calibration and the validation datasets were calculated to assess model performance. These randomized investigations generated a distribution of fit statistics allowing for the evaluation of model stability, as well as uncertainty in the model predictions. The strength contribution of PLSR loadings by individual wavelengths was also assessed using the VIP selection statistics, which highlight the importance of individual wavelengths in explaining the variation in both the response and predictor variables: larger weightings confer higher value to the contribution of individual wavelengths to the predictive model [12,63]. The modeling approach and the data analyses were run using the ‘pls’ package in R (www.r-project.org; [64]).

Before developing the final modeling, we investigated preliminary models to identify poorly predicted outliers. Prediction residuals were explored to identify potential outliers [14]. Spectral profiles of outliers were further examined for errors (e.g., elevated reflectance in the VIS wavelengths, spectral jumps produced by misaligned detector splicing, concave spectral shape at the red-edge peak) all likely due to the operational errors during spectral measurements (in reference or target collections). The standard measurements of the outliers were also examined for extremes in the data distribution. The outliers removed accounted for approximately 10% of the initial data, which was in agreement with previous studies (e.g., [14,17]).

We also performed an external validation by applying PLSR coefficients on a dataset independent from the one used for calibration and validation (ca. 20% of the whole dataset). Relations between predicted and observed values were tested by regression analysis, and fit statistics (i.e., *R^2^*, RMSE, bias) were investigated to assess the model estimation accuracy.

### 4.5. Estimation of Leaf Traits by Vegetation Indices and PLSR Models

Three common VIs were calculated from spectra: the PRI = (R_531_ − R_570_)/(R_531_ + R_570_) [39], scaled as sPRI = (PRI + 1)/2 to avoid negative values, was determined to assess any potential effect on the photochemical system; the PSRI = (R_678_ − R_500_)/R_750_ [65], scaled as sPSRI = (PSRI + 1)/2 to avoid negative values, was determined to evaluate the occurrence of any potential senescence process; and the CI = (R_750_ − R_705_)/(R_750_ + R_705_) [11] was determined to investigate any potential variation in chlorophyll content. R_x_ indicates the reflectance at x nm wavelength. 

Chlorophyll *a* fluorescence parameters were estimated from spectra by using the best performing PLSR models. As stated above, vegetation indices and spectra-derived ChlF parameters were calculated from the spectra averaged per plant.

### 4.6. Statistical Analyses for the Effects of Ozone on Leaf Traits Derived from Spectra, and on the Leaf Spectral Signatures (i.e., Hyperspectral Phenotyping)

The Shapiro–Wilk test was first used to assess the normal distribution of spectral indices and ChlF parameters derived from the spectra by the PLSR-models. The effect of O_3_ on these leaf traits (the statistical unit was the single plant) was then investigated by a one-way repeated measure analysis of variance (ANOVA). Tukey’s test was used as post-hoc test. Statistically significant effects were considered for *p* < 0.05. Univariate statistical analyses were run in JMP 13.2.0 (SAS Institute Inc., Cary, NC, USA).

The effects of O_3_ on the untransformed reflectance profiles (averaged per plant) were assessed by PERMANOVA [66], employing Euclidian measurements of dissimilarity and 10,000 permutations; and PCoA was used on these spectral data to visualize the spectral responses to O_3_, always using Euclidean distances through the ‘vegan’ package in R (www.r-project.org; [67]). Finally, the capability of spectroscopy to classify O_3_ treatments was further determined by PLS-DA [8,68]. The analyses were run 500 times by splitting observations into different groups of calibration (training) and validation (testing) sets, and the number of correct classifications both in the calibration and in the validation sets across the 500 iterations was used to determine the accuracy of the tested model. The calibration:validation data ratio and the number of components used to get the models that would give the best fit to the data were determined by iteratively running the PLS-DA models with different calibration:validation data ratios (i.e., 50:50, 70:30, 80:20), and the numbers of components, and were based on the highest kappa values returned for the validation models. Partial least squares-discriminant analysis was run with the ‘caret’ and ‘vegan’ packages in R (www.r-project.org; [67,69]).

## Figures and Tables

**Table 1:** Range of wavelengths, number of components (Com), model goodness-of-fit (*R^2^*), root mean square error (RMSE), and percent RMSE of the data range (%_RMSE_) for calibration (Cal) and validation (Val) data generated using 500 random permutations of the data with 80% used for Cal and 20% used for the Val for the PLSR models predicting chlorophyll *a* fluorescence parameters from spectra of date palm leaves. Bias outputs are not shown as they were always lower than 0.01 for both Cal and Val. Data are shown as mean ± standard deviation. Parameters: F_0_, minimum fluorescence yield in the dark-adapted state; F_m_, maximum fluorescence yield in the dark-adapted state; F_v_/F_m_, maximum quantum efficiency of PSII photochemistry; F_s_, steady state fluorescence intensity in light-adapted state; F_m_’, maximum fluorescence intensity in the light-adapted state; F_0_′, minimum fluorescence intensity in the light-adapted state; Φ_PSII_, PSII operating efficiency in light conditions; F_v_’/F_m_’, maximum efficiency of PSII in light conditions; ETR, electron transport rate (µmol m^−2^ s^−1^); qP, photochemical quenching; qN, non-photochemical quenching calculated as (F_m_ − F_m_’)/(F_m_ − F_0_′); NPQ, non-photochemical quenching calculated as (F_m_ − F_m_’)/F_m_’; qL, fraction of open PSII centers; P, fraction of light absorbed in PSII antennae that is utilized in PSII photochemistry; D, fraction of light absorbed in PSII antennae that is dissipated thermally. See Section 4.2 for parameter calculations.

Parameter	Range (nm)	Com	Cal			Val		
			*R^2^*	RMSE	%_RMSE_	*R^2^*	RMSE	%_RMSE_
**F_0_**	600–900	8	0.69 ± 0.03	0.01 ± 0.00	13	0.53 ± 0.14	0.01 ± 0.00	17
**F_m_**	600–900	7	0.74 ± 0.03	0.04 ± 0.00	12	0.60 ± 0.15	0.05 ± 0.01	15
**F_v_/F_m_**	600–900	8	0.85 ± 0.02	0.04 ± 0.00	11	0.77 ± 0.08	0.04 ± 0.01	11
**F_s_**	400–1200	4	0.46 ± 0.04	0.01 ± 0.00	17	0.39 ± 0.18	0.02 ± 0.00	18
**F_m_’**	400–1200	8	0.80 ± 0.03	0.03 ± 0.00	9	0.65 ± 0.14	0.04 ± 0.01	12
**F_0_′**	600–900	7	0.47 ± 0.05	0.01 ± 0.00	17	0.21 ± 0.19	0.01 ± 0.00	21
**Φ_PSII_**	600–900	10	0.87 ± 0.03	0.03 ± 0.00	8	0.73 ± 0.12	0.04 ± 0.01	11
**F_v_’/F_m_’**	600–900	7	0.84 ± 0.02	0.04 ± 0.00	9	0.75 ± 0.10	0.04 ± 0.01	11
**ETR**	400–2400	12	0.93 ± 0.01	2.63 ± 0.15	6	0.82 ± 0.08	4.31 ± 0.68	10
**qP**	600–900	11	0.87 ± 0.02	0.02 ± 0.00	9	0.68 ± 0.14	0.04 ± 0.01	14
**qN**	600–900	7	0.70 ± 0.04	0.05 ± 0.00	12	0.53 ± 0.17	0.06 ± 0.01	15
**NPQ**	600–900	6	0.40 ± 0.05	0.11 ± 0.00	16	0.26 ± 0.19	0.12 ± 0.02	18
**qL**	600–900	8	0.86 ± 0.02	0.04 ± 0.00	8	0.76 ± 0.12	0.05 ± 0.01	11
**P**	400–2400	12	0.93 ± 0.01	0.02 ± 0.00	6	0.81 ± 0.08	0.04 ± 0.01	10
**D**	600–900	7	0.84 ± 0.02	0.04 ± 0.00	7	0.76 ± 0.11	0.04 ± 0.01	10

## Supplementary Materials

Supplementary materials can be found at https://www.mdpi.com/1422-0067/21/17/6441/s1.

## Abbreviations

AA	Ambient air
ANOVA	Analysis of variance
AOT40	Accumulated exposure over a threshold of 40 ppb
ChlF	Chlorophyll *a* fluorescence
CI	Chlorophyll index
D	Fraction of light absorbed in PSII antennae that is dissipated thermally
EO	Elevated ozone
ETR	Electron transport rate
F_0_	Minimum fluorescence yield in the dark-adapted state
F_0_′	Minimum fluorescence intensity in the light-adapted state
F_m_	Maximum fluorescence yield in the dark-adapted state
F_m_’	Maximum fluorescence intensity in the light-adapted state
F_s_	Steady state fluorescence intensity in light-adapted state
F_v_/F_m_	Maximum quantum efficiency of PSII photochemistry
F_v_’/F_m_’	Maximum efficiency of PSII in light conditions
FACE	Free air controlled exposure
MO	Moderate ozone
NDVI	Normalized difference vegetation index
NIR	Near-infrared spectral region
NPQ	Non-photochemical quenching calculated as (F_m_-F_m_’)/F_m_’
O_3_	Ozone
P	Fraction of light absorbed in PSII antennae that is utilized in PSII photochemistry
PAM	Pulse-amplitude-modulation
PCoA	Principal coordinates analysis
PERMANOVA	Permutational analysis of variance
PLS-DA	Partial least squares discriminant analysis
PLSR	Partial least squares regression
PRESS	Predicted residual sum of squares
PRI	Photochemical reflectance index
PSI	Photosystem I
PSII	Photosystem II
PSRI	Plant senescence reflectance index
qL	Fraction of open PSII centers
qN	Non-photochemical quenching calculated as (F_m_-F_m_’)/(F_m_-F_0′_)
qP	Photochemical quenching
*R* ^2^	Model goodness-of-fit
RMSE	Root mean square error
sPRI	Scaled photochemical reflectance index
sPSRI	Scaled plant senescence reflectance index
SWIR	Short-wave infrared spectral region
VIs	Vegetation indices
VIP	Variable importance to the projection
VIS	Visible spectral region
Φ_PSII_	PSII operating efficiency in light conditions
%_RMSE_	Percent root mean square error of the data range
