# Supplementary material for: Hyperspectral Reflectance of Light-Adapted Leaves Can Predict Both Dark- and Light-Adapted Chl Fluorescence Parameters, and the Effects of Chronic Ozone Exposure on Date Palm (Phoenix dactylifera)
Source: Int J Mol Sci. 2020 Sep 3;21(17):6441. doi: 10.3390/ijms21176441 (PMC7504383; doi:10.3390/ijms21176441)
Supplement: Supplementary file 1 [file ijms-21-06441-s001.pdf]

International Journal of *Molecular Sciences*

MDPI

Supplementary Material

Table S1. Tested wavelength ranges of (i) preliminary PLSR-models used for the prediction of Chl fluorescence parameters from spectra of date palm leaves, and (ii) preliminary permutational multivariate analysis of variance (PERMANOVA) for the effects of ozone on reflectance profiles of date palm leaves. Final ranges are underlined. Parameters: F_0, minimum fluorescence yield in the dark-adapted state; F_m, maximum fluorescence yield in the dark-adapted state; F_v/F_m, maximum quantum efficiency of PSII photochemistry; F_s, steady state fluorescence intensity in light-adapted state; F_m', maximum fluorescence intensity in the light-adapted state; F_0', minimum fluorescence intensity in the light-adapted state; Φ_{PSII}, PSII operating efficiency in light conditions; F_v'/F_m', maximum efficiency of PSII in light conditions; ETR, electron transport rate (μmol m^{-2} s^{-1}); qP, photochemical quenching; qN, non-photochemical quenching calculated as $(F_m-F_m')/(F_m-F_0')$; NPQ, non-photochemical quenching calculated as $(F_m-F_m')/F_m'$; qL, fraction of open PSII centres; P, fraction of light absorbed in PSII antennae that is utilized in PSII photochemistry; D, fraction of light absorbed in PSII antennae that is dissipated thermally. See *subsection 4.2* for parameter calculations.

Analysis	Parameter	Tested wavelength ranges (nm)
**PLSR**	**F_0**	400-2400, 950-2400, 1400-2400, 400-900, 400-800, 400-700, 400-1200, 500-900, 600-900, 450-1000
	**F_m**	400-2400, 950-2400, 1400-2400, 400-900, 400-800, 400-700, 400-1200, 500-900, 600-900, 450-1000
	**F_v/F_m**	400-2400, 950-2400, 1400-2400, 400-900, 400-800, 400-700, 400-1200, 500-900, 600-900, 450-1000
	**F_s**	400-2400, 950-2400, 1400-2400, 400-900, 400-800, 400-700, 400-1200, 500-900, 600-900, 450-1000
	**F_m'**	400-2400, 950-2400, 1400-2400, 400-900, 400-800, 400-700, 400-1200, 500-900, 600-900, 450-1000
	**F_0'**	400-2400, 950-2400, 1400-2400, 400-900, 400-800, 400-700, 400-1200, 500-900, 600-900, 450-1000
	**Φ_{PSII}**	400-2400, 950-2400, 1400-2400, 400-900, 400-800, 400-700, 400-1200, 500-900, 600-900, 450-1000
	**F_v'/F_m'**	400-2400, 950-2400, 1400-2400, 400-900, 400-800, 400-700, 400-1200, 500-900, 600-900, 450-1000
	**ETR**	400-2400, 950-2400, 1400-2400, 400-900, 400-800, 400-700, 400-1200, 500-900, 600-900, 450-1000
	**qP**	400-2400, 950-2400, 1400-2400, 400-900, 400-800, 400-700, 400-1200, 500-900, 600-900, 450-1000
	**qN**	400-2400, 950-2400, 1400-2400, 400-900, 400-800, 400-700, 400-1200, 500-900, 600-900, 450-1000
	**NPQ**	400-2400, 950-2400, 1400-2400, 400-900, 400-800, 400-700, 400-1200, 500-900, 600-900, 450-1000
	**qL**	400-2400, 950-2400, 1400-2400, 400-900, 400-800, 400-700, 400-1200, 500-900, 600-900, 450-1000
	**P**	400-2400, 950-2400, 1400-2400, 400-900, 400-800, 400-700, 400-1200, 500-900, 600-900, 450-1000
	**D**	400-2400, 950-2400, 1400-2400, 400-900, 400-800, 400-700, 400-1200, 500-900, 600-900, 450-1000
**PERMANOVA**	**-**	400-2400, 950-2400, 1400-2400, 400-900, 400-800, 400-700, 400-1200, 500-900, 450-1000, 1800-2400, 700-1200

Table S2. Averaged model goodness-of-fit (R^2) and root mean square error (RMSE) for external validation generated with PLSR-models predicting Chl fluorescence parameters from spectra of date palm leaves. Bias outputs are not shown as always lower than 0.01. Parameters: F_0, minimum fluorescence yield in the dark-adapted state; F_m, maximum fluorescence yield in the dark-adapted state; F_v/F_m, maximum quantum efficiency of PSII photochemistry; F_m', maximum fluorescence intensity in the light-adapted state; Φ_{PSII}, PSII operating efficiency in light conditions; F_v'/F_m', maximum efficiency of PSII in light conditions; ETR, electron transport rate (μmol m^{-2} s^{-1}); qP, photochemical quenching; qN, non-photochemical quenching calculated as $(F_m-F_m')/(F_m-F_0')$; qL, fraction of open PSII centres; P, fraction of light absorbed in PSII antennae that is utilized in PSII photochemistry; D, fraction of light absorbed in PSII antennae that is dissipated thermally. See *subsection 4.2* for parameter calculations.

Param	R^2	RMSE
**F_0**	0.54	0.01
**F_m**	0.02	0.02
**F_v/F_m**	0.73	0.04
**F_m'**	0.04	0.02
**Φ_{PSII}**	0.77	0.05
**F_v'/F_m'**	0.31	0.03
**ETR**	0.76	5.49
**qP**	0.59	0.06
**qN**	0.55	0.05
**qL**	0.13	0.01
**P**	0.74	0.05
**D**	0.86	0.04

Table S3. Summary of confusion matrix results indicating proportion of classification accuracies, and misclassifications, for individual ozone treatments on date palm leaves. Validation data generated using 80% of the data for calibration and 20% for validation are reported. Each row represents the correct (bolded) and incorrect (nonbolded) classifications for a specific ozone treatment. Values are mean of 500 permutations of partial least squares discriminate analysis.

	AA	MO	EO
**AA**	**0.74**	0.22	0.04
**MO**	0.10	**0.76**	0.14
**EO**	0.01	0.07	**0.92**
